# Segmentally homologous neurons acquire two different terminal neuropeptidergic fates in the *Drosophila* nervous system

**DOI:** 10.1371/journal.pone.0194281

**Published:** 2018-04-10

**Authors:** Hugo Gabilondo, Irene Rubio-Ferrera, María Losada-Pérez, Delia del Saz, Yolanda León, Isabel Molina, Laura Torroja, Douglas W. Allan, Jonathan Benito-Sipos

**Affiliations:** 1 Departamento de Biología, Universidad Autónoma de Madrid, Cantoblanco, Madrid, Spain; 2 Department of Cellular and Physiological Sciences, University of British Columbia, Vancouver, British Columbia, Canada; Biocenter, Universität Würzburg, GERMANY

## Abstract

In this study, we identify the means by which segmentally homologous neurons acquire different neuropeptide fates in *Drosophila*. Ventral abdominal (Va)-neurons in the A1 segment of the ventral nerve cord express DH31 and AstA neuropeptides (neuropeptidergic fate I) by virtue of *Ubx* activity, whereas the A2-A4 Va-neurons express the Capa neuropeptide (neuropeptidergic fate II) under the influence of *abdA*. These different fates are attained through segment-specific programs of neural subtype specification undergone by segmentally homologous neurons. This is an attractive alternative by which Hox genes can shape *Drosophila* segmental neural architecture (more sophisticated than the previously identified binary “to live” or “not to live” mechanism). These data refine our knowledge of the mechanisms involved in diversifying neuronal identity within the central nervous system.

## Introduction

The ability to direct cell subtype specification is essential for the use of stem cells to produce clinically relevant advances in disease treatment. To enable routine generation of specific neurons to replace those that are lost or damaged, it is essential to fully understand the mechanisms of neuronal subtype specification. An interesting feature of nervous system development is the emergence of characteristic neural subtypes in a segment-specific manner, under the control of Hox genes. Studies in *Drosophila melanogaster* continue to provide fundamental insights into this process [1–3]. In the developing *Drosophila* ventral nerve cord (VNC), a serially homologous set of 30 neural stem cells termed neuroblasts (NBs) arise in the three thoracic segments (T1-T3) and in the eight abdominal segments (A1-A8) [3]. Segments A9 and A10 have fewer neuroblasts [2].

Despite this segmental repetition of homologous NBs, there are segment-specific differences in neuron number and terminal identity, all driven in a Hox-dependent manner. First, there are Hox-dependent differences in NBs cell cycle exit and programmed cell death (PCD) throughout lineage progression, which accounts for the increase in thoracic vs. abdominal lineages of certain NBs [1, 4, 5]. Second, the type of lineage progression can differ in a Hox-dependent manner; NBs 1–1 (NB1-1) is a NBs in thoracic segments and a neuroglioblast in abdominal segments, whereas NB6-1 is a neuroglioblast in thoracic and a glioblast in abdominal segments [6]. Third, homologous postmitotic neurons can be generated throughout the anterior-posterior (AP) axis, but in specific segments can subsequently undergo Hox-dependent PCD [7–9] [10] or a delay in terminal differentiation of homologous neurons [11, 12]. These last mechanisms act in postmitotic neurons to alter survival or the timing of differentiation, but not the terminal identity itself.

Here we describe an alternative to the third diversification mechanism, in which developmentally homologous neurons attain segment-specific differences in terminal identity, their neuropeptidergic identity. Previous work showed that a single ventral abdominal (Va)-neuron is born in each A1-A8 hemisegment and that all display the same set of markers (Dimm and Dac) shortly after terminal mitosis, in embryonic stage 15 embryos (Stg 15). Va-neurons generated in abdominal segments A5-A8 are eliminated by PCD [8, 9]. Va generated in A2-A4 segments express the Capa neuropeptide, whereas the Va born in A1 express the pro-neurosecretory transcription factor Dimmed (Dimm); it is nonetheless unclear whether the Va-A1 cell finally differentiates into a peptidergic neuron and, if so, which neuropeptide gene it expresses.

In this study we report the neuropeptidergic nature of the Va-A1 cell, which expresses DH31 (Diuretic hormone 31) and AstA (Allatostatin A) neuropeptides (neuropeptidergic fate I) whereas the A2-A4 Va-neurons express the Capa neuropeptide (neuropeptidergic fate II). We are unaware of previous descriptions of segmentally homologous neurons that acquire distinct neuropeptide fates. We describe the role of segmental Hox gene activity in determining these differences in terminal identity. Our data demonstrate that developmentally homologous neurons undergo diverse neuropeptidergic differentiation programs in a segment-specific manner and that the segmental Hox genes execute those programs.

## Results

### The orphan-identity Va Dimmed-positive neuron of the first abdominal segment acquires a DH31/AstA peptidergic fate

**Fig 1:**
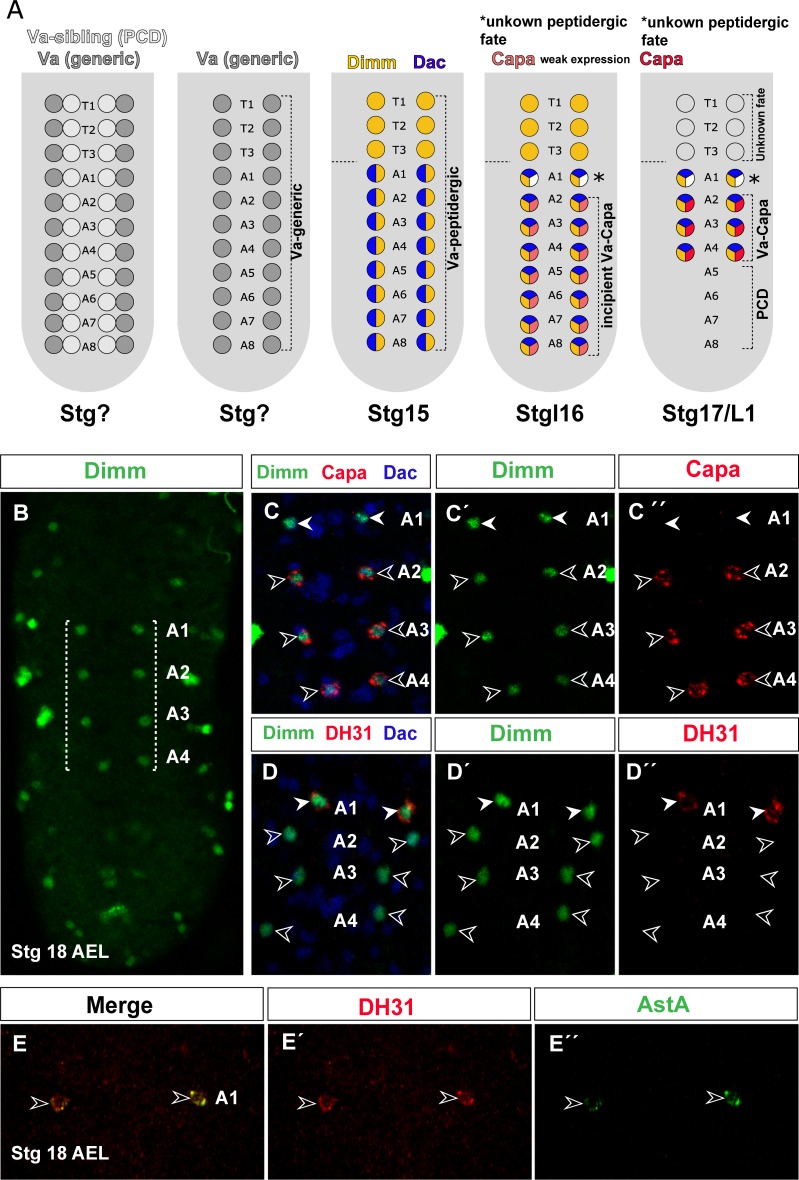
Identification of Diuretic hormone 31 (DH31) and Allatostatin A (AstA) neuropeptides as Va-A1 terminal markers. (A) Model of the segmentally synonymous Va-neurons [8, 9]. In A1, the Va neuron fate is unknown. (B) Dimm expression marks Va-neurons in four abdominal segments (A1 to A4). (C) Overlap of Capa (red), Dimm (green), and Dac (blue) at Stg 18 h (after egg laying; AEL) in wild type. Capa is expressed in A2-A4 abdominal segments, but not in Va-A1. (D) Overlap of DH31 (red), Dimm (green), and Dac (blue) at Stg 18 in wild type. The DH31 neuropeptide is expressed in Va-A1 neurons. (E) Overlap of DH31 (red) and AstA (green) at Stg 18 h AEL in wild type, showing that AstA is also expressed in Va-A1 neurons. Genotype: *OregonR*.

Previous studies showed that within all VNC segments, a single pair of Va neurons emerges per segment from NB5-3 within a *castor* (*cas*) temporal window (Fig 1A) [8, 9]. These cells are easily identified by their medial position and expression of the pro-neurosecretory transcription factor Dimm at Stg 15. Thereafter these neurons undergo divergent, segment-specific differentiation programs. In thoracic segments, Dimm expression is extinguished and the neuron fate is completely unknown. In abdominal segments, all Va neurons express Dimm and Dachschund (Dac), but are otherwise not alike. In A2-A4, Va neurons express the Capability (Capa) neuropeptide (henceforth Va-Capa neurons; also ABCA) [9]) (Fig 1A and 1C). In A5-A7, Va-neurons undergo segment-specific PCD shortly after initiation of Capa expression (Fig 1A; [8, 9]. Uncertainty nonetheless shrouds the neuron born in the first abdominal segment (A1). This cell robustly expresses the Dimm transcription factor, although it never expresses the Capa neuropeptide (Fig 1A–1C) [8]. Indeed, it remains unclear whether the Va-A1 cell finally differentiates into a peptidergic neuron and if so, which neuropeptide gene it expresses. Here we focus on this intriguing neuron born in the first abdominal segment.

Previous research suggested that the Va-A1 neuron does not undergo PCD, since Capa expression is not rescued on genetic backgrounds in which apoptosis is inhibited [8, 9]. These findings can be explained in two ways, either (1) the Va-A1 neuron is not programmed to express the Capa neuropeptide and dies by PCD, or (2) the Va-A1 neuron is not programmed to express the Capa neuropeptide and acquires another fate. In both cases, rescue of Capa is impossible when PCD is abolished.

To rule out the first possibility, we monitored the Va-A1 cell throughout its development for coexpression and position of Dimm/Dac (which unequivocally label Va cells). We found that Va-A1 cells are present at least until the third larvae instar (S1 Fig); this coincides with the absence of caspase expression in these cells at Stg 15, when their A5-A7 cell counterparts undergo apoptosis and express the caspase apoptotic marker [8]. These results suggest that these cells are functional at larval stages, and would thus indicate that homologous neurons can differentiate to two distinct neuropeptidergic fates. Nonetheless, the neuropeptide expressed by Va-A1 neurons, if any, has not been described.

To identify and define the putative neuropeptide expressed by these cells, we conducted a co-immunostaining study of Va-A1 cells (Dimm/Dac-positive) using selected neuropeptide antibodies based on neuropeptide expression in the *Drosophila* VNC [13]. We found that the Va neurons generated in the A1 segment express not one but two neuropeptides, Diuretic hormone 31 (DH31) and Allatostatin A (AstA) (Fig 1D and 1E); we thus denominated these cells Va-DH31 neurons. This Va neuron coincides with an abdominal and ventral neuron also born in the A1 segment and that also expresses DH31 and AstA neuropeptides [13]. These authors nonetheless describe a Capa-expressing neuron born in the same segment, which is incompatible with other findings [8, 9]; indeed, the Taghert group report Capa-positive cells in segments A1-A3 [13], whereas previous studies place them from A2 to A4 [8, 9].

The Va generated in the A1 segment thus expresses both DH31 and AstA neuropeptides. We subsequently used either DH31 or AstA antibodies, depending on compatibility with other antibodies; when possible, we used both and obtained similar results.

### Va-DH31 and Va-Capa neurons are produced by segmentally homologous NB in the same temporal window

**Fig 2:**
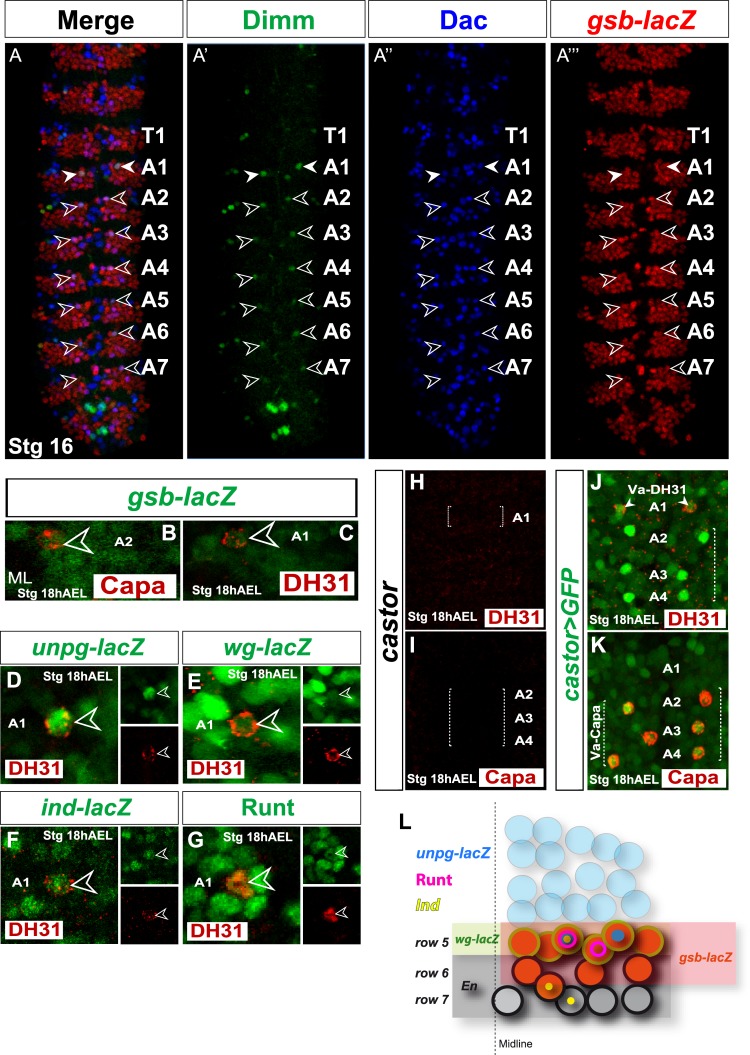
Va-DH31 and Va-Capa are segmentally homologous neurons generated by the NB5-3 in a cas temporal window. (A) Overlap of *gsb-lacZ* (red), Dimm (green), and Dac (blue) at Stg 16 in a wildtype VNC. All Va neurons from A1 to A7 show a medial anterior position in the *gsb-Z* pattern of every segment. (B-C) Overlap of *gsb-lacZ* (green), with Capa and DH31 (red in B and C, respectively) at Stg 18 h AEL in a wildtype VNC. Both Va-Capa (B) and Va-DH31(C) cells show a similar medial anterior position in the *gsb-Z* pattern. (D-G) Overlap of DH31 (red) and various progenitor NB markers (green): *unpg*-*lacZ* (D), *wg*-*lacZ* (E), *ind*-*lacZ* (F) and Runt (G). DH31 neurons overlap all of them, which suggests they are generated by the NB5-3. (H-I) DH31 (H) and Capa (I) expression in *cas* mutants (*cas*^*Δ1*^/*cas*^*Δ1*^). (J-K) Overlap of DH31 (J) and Capa (K) with *cas-Gal4>UAS gfp*. *castor-Gal4* is active in both Va-DH31 and Va-Capa neurons. ML, midline; anterior is up in all images.

Va cells are generated along the VNC in a distinctive pattern easily identified by their ventral localization. Dimm/Dac marker co-expression is used to validate their identity. To confirm that Va-DH31 neurons were raised from the same neuroblasts as Va-Capa neurons, we analyzed this cell in detail. We studied Va-DH31 neuron location within the *gooseberry-lacZ* (*gsb-lacZ*) pattern, and found that Va cells always had a precise medial and anterior position in the *gsb-Z* pattern of each segment, including A1 (Fig 2A–2C). With the same set of genetic markers used to identify the progenitor NB of the Va-Capa cell [9], we determined that Va-DH31 arises from NB5-3 (Fig 2D–2G; summary in Fig 2L). We thus concluded that both Va-Capa and Va-DH31 arose from the same progenitor NB.

Since Va-Capa neurons are generated during the *castor* (*cas*) temporal window [9], we tested whether Va-DH31 also originated in this window. Analysis of DH31 expression in *cas* mutant embryos showed complete absence of DH31 and Capa signals (Fig 2H and 2I). In addition, we found that the Va-DH31 neuron expressed *cas-Gal4>UAS-GFP* (Fig 2J and 2K), as described for Va-Capa neurons [9]. The results indicate that both Va-Capa and Va-DH31 neurons are generated in the *cas* temporal window.

Our data show that Va-DH31 and Va-Capa neurons are generated from the same NB during identical temporal windows, and both occupy the same position within the *gsb-lacZ* pattern. These results strongly suggest that they are segmentally homologous neurons generated in distinct segments, which acquire distinct neuropeptide fates–Capa in Va neurons from segments A2-A4 and DH31/AstA in the Va-A1 cell.

### Ultrabithorax directs A1-specific differentiation in Va-DH31 neurons

The role of Hox genes in segmentally homologous Va neurons has been analyzed [8], although in that study the only available terminal marker for the Va-A1 neuron was the pro-neurosecretory transcription factor Dimm. Identification of DH31/AstA as terminal markers of the Va-A1 neuron allowed us to study the role of Hox patterning factors in segment-specific diversification of Va-neurons and to confirm the generic Dimm results.

**Fig 3:**
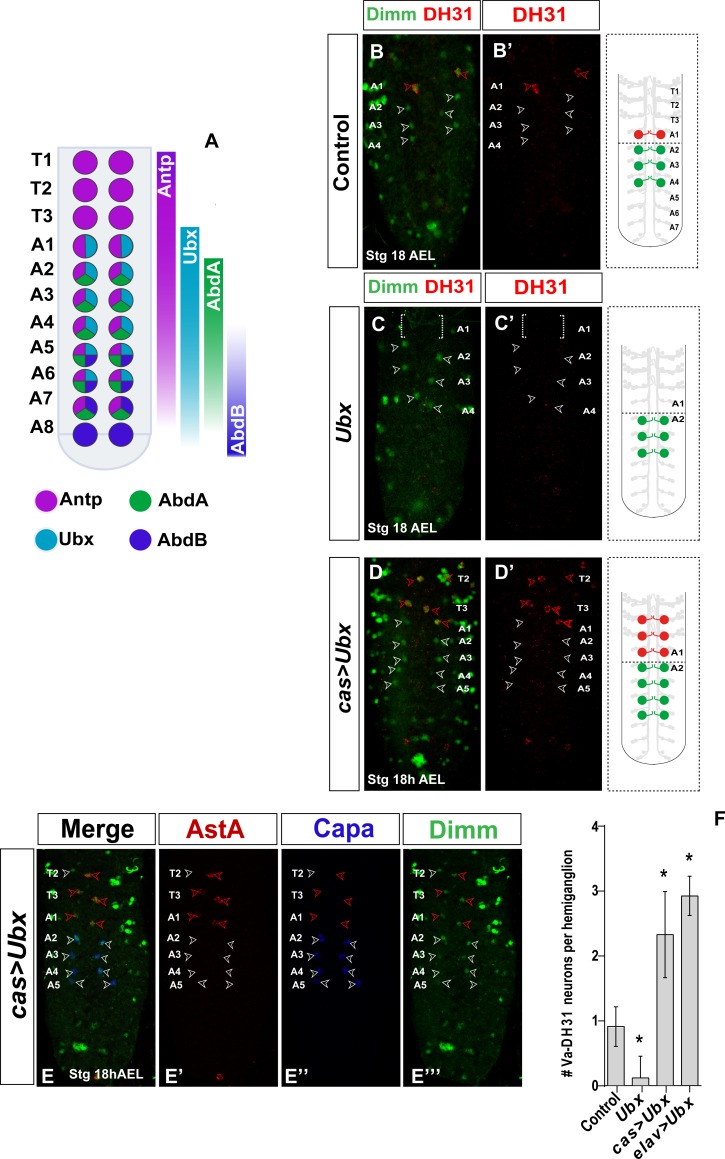
*Ultrabithorax* induces DH31 expression. (A) Scheme summarizing Hox expression in Va-neurons (modified from[8]). Expression of Dimm (green) and DH31 (red) in *Ubx* mutants (B) and a *cas>Ubx* background (C). Dimm and Dac expression is lost in *Ubx* mutants, whereas *Ubx* misexpression results in anterior expansion of DH31, Dimm and Dac expression into Va-T2-T3 neurons. (D) Expression of AstA (red), Capa (blue), and Dimm (green) on a *cas>Ubx* background. Note posterior expansion of Va-Capa neurons into Va-A5 neurons. (E) Quantitation of these genetic studies [*n* ≥8 VNC in all genotypes; asterisks indicate significant difference compared with controls (Student’s *t*-test, *P* <0.001)]. Genotypes: (B) *Ubx*^*1*^/*Ubx*^*1*^, (C-D) *cas-Gal4/UAS-Ubx*, (E) *OregonR*, *Ubx*^*1*^/*Ubx*^*1*^, *cas-Gal4/UAS-Ubx*, *elav-Gal4/UAS-Ubx*.

We tested whether Ubx had a role in Va neuron acquisition of their final peptidergic fate. Previous studies showed that Dimm expression in Va-A1 neurons depends on the Hox factor Ultrabithorax (Ubx), although the lack of specific markers for complete Ubx maturation prevented conclusive analysis of its late final fate. We found that DH31 was lost on a null *Ubx* mutant background (Fig 3B) and, in accordance with the earlier report, Dimm expression was also lost (Fig 3B) [8]. To verify a *Ubx* role as a critical factor in Va-DH31 vs Va-Capa differentiation, we misexpressed *Ubx* in all Va neurons, using *castor-Gal4* (expressed in the NB that gives rise to Va neurons and in Va neurons themselves) [9] and *elav-Gal4* (primarily a post-mitotic neuron-specific driver, which initiates low expression at early time points in certain lineages) [14]. Using these *Gal4* drivers, we observed the same phenotype; *Ubx* misexpression produced anterior expansion of DH31-expressing Va neurons in thoracic segments T2-T3, a location appropriate for Va neurons (Figs 3C, 3E and S2A). The T2-T3 Va-neurons also expressed Dimm and Dac, indicative of a full T1-T3 Va neuron transformation to an A1-type Va neuron. Ubx is thus necessary in Va-A1 neurons to induce Dimm as well as DH31/AstA expression, and is sufficient to induce Va-A1-type terminal differentiation in T2-T3 Va neurons.

The PCD undergone by the Va-Capa neurons in segments A5-A7 is triggered by the Hox factor Abd-B [8]. When *Ubx* was misexpressed, however, we found posterior expansion of Va-Capa neurons in abdominal segment A5, which appears to abort the PCD program (Fig 3D and 3E). Hox genes can be co-expressed in single cells, mostly around the borders of their expression domains [7, 8]. The posterior Hox gene normally suppresses anterior Hox gene function through transcriptional repression [15] and/or phenotypic suppression [16]. These data offer another example of an anterior Hox gene acting dominantly over a posterior Hox gene; in our case, *Ubx* overexpression interferes with posterior *Abd-B* function late in development.

These results establish a critical role for Ubx in Va-A1 cell differentiation to DH31/AstA- vs Capa-expressing neurons.

### Abdominal-A represses Va-DH31 fate and determines Va-Capa neurons

**Fig 4:**
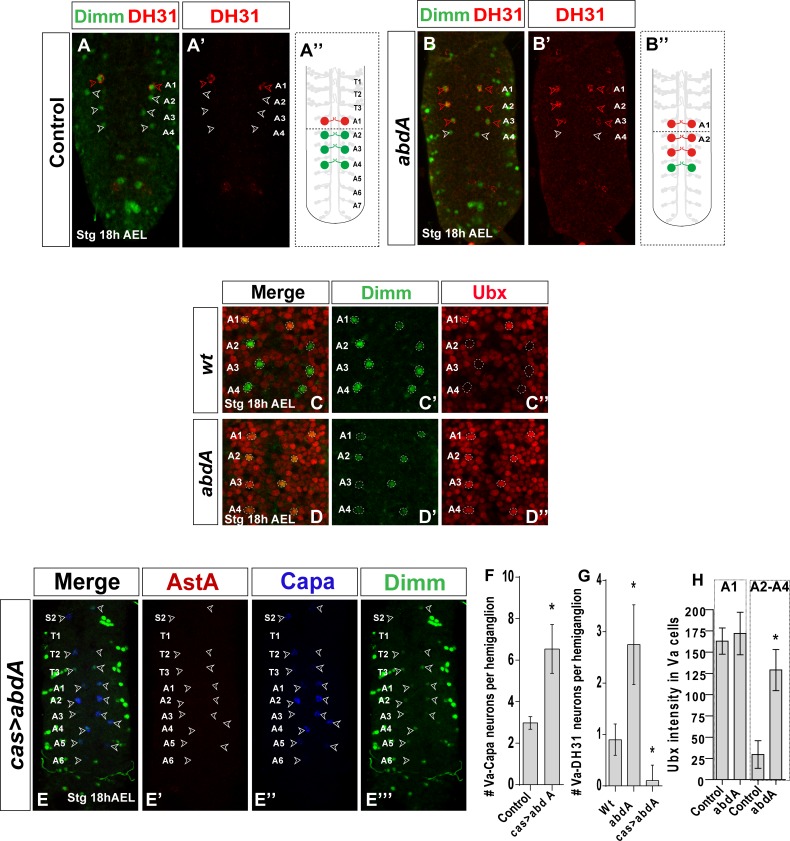
Postmitotic *abdominal-A* ignores posterior prevalence in Va-neurons. (A) Expression of Dimm (green) and DH31 (red) shows that Va-Capa neurons are transformed into Va-DH31 neurons in *abdA* mutants. (B-C) Expression of Dimm (green) and Ubx (red) in control (B) and *abdA* mutants (C). Ubx expression is increased in A2-A4 Va-neurons. (D) Expression of AstA (red), Capa (blue), and Dimm (green) in a *cas>abdA* background. Va-DH31 fate was expanded anteriorly and Va-neurons in A5 and A6 do not undergo PCD. (E-G) Quantitation of genetic studies [*n* ≥8 VNC in all genotypes; asterisks indicate significant difference compared with controls (Student’s *t*-test, *P*<0.001)] Genotypes: (A, C) *abdA*^*MX1*^/*abdA*^*MX1*^, (B) *OregonR*, (D) *cas-Gal4*/*UAS-abdA*.

Capa expression is lost in A2-A3 in *abdA* mutant Va-neurons although Dimm/Dac expression is normal [8], which implied the need for *abdA* in Va-Capa neuron differentiation in A2-A4. To determine whether this phenotype is associated with an A2-A3 Va-neuron identity switch to A1-type, we tested whether Va-Capa neurons in A2-A3 are transformed into Va-DH31 neurons on an *abdA* mutant background. We found that these cells differentiated into Va-DH31-type neurons (Fig 4A and 4F). To ascertain whether this posterior expansion of Va-DH31 fate was produced by a posterior Ubx increase, we analyzed Ubx protein levels on an *abdA* mutant background and indeed found Ubx expression in A2-A4 Va-neurons (Fig 4B, 4C and 4G). Despite the high Ubx level and the absence of *abdA* (Fig 4C), in some cases the A4 Va neuron maintains Capa expression [8].

We expressed *abdA* ectopically, again using *elav-Gal4* and *cas-Gal4* to drive gene expression in all Va neurons, to determine whether expression outside its typical domain is sufficient for Va-DH31-type repression and Va-Capa-type differentiation. We observed Va-Capa neurons in T1-A1 segments in *cas-Gal4>UAS-abdA* embryos (Fig 4D and 4E). Results were variable with the *elav-Gal4* driver. In some cases, Va-DH31 fate expanded anteriorly, with DH31 expression in Va-T1-T3 neurons (S2B Fig), although it was not fully penetrant [8]. We also found that *abdA* misexpression rescued Va-neurons in A5 and A6 from PCD, which is normally determined by posterior-acting *Abd-B* (Fig 4D and 4E). It thus appears that AbdA determines Va-Capa fate, and when overexpressed overrides anterior and posterior Hox gene function.

### Va-DH31 fate is repressed by Antennapedia in thoracic segments

In thoracic segments, Dimm expression disappears from Va neurons at late Stg 15. These cells are no longer evident within the thoracic segments, and their fate remains a puzzle [8] (Fig 1A). The loss of Dimm expression at late Stg 15 in T1-T3 segments, shortly after generation of these neurons, led us to postulate that high Antennapedia (Antp) levels have a late role in suppressing Va-neuron terminal differentiation to Va-A1.

**Fig 5:**
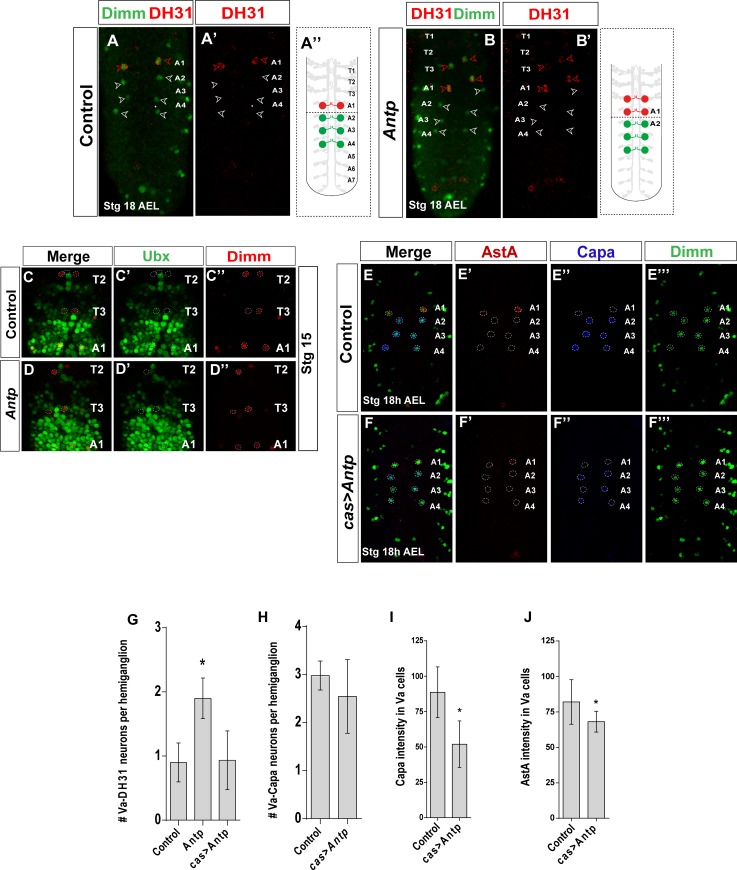
*Antennapedia* shows phenotypic dominance over the co-expressed *Ultrabithorax* in Va-T3 neurons. (A) Expression of DH31 (red) and Dimm (green) showing that T3 Va-neurons expressed DH31 in *Antp* mutants. (B-C) Expression of Ubx (green) and Dimm (red) in *Antp* mutants (B) and controls (C) at Stg 15. Ubx is normally expressed at low level in T3 Va-neurons at Stg 15; this expression is unaffected in *Antp* mutants. (D-E) Expression of AstA (red), Capa (blue), and Dimm (green) in controls (D) and a *cas>Antp* background (E). Note that Capa expression (but not DH31/AstA) is downregulated when *Antp* is overexpressed. (F) and (G) Quantitation of these genetic studies [*n*≥11 VNC in all genotypes; asterisks indicate significant difference compared with control (Student’s *t*-test, *P*<0.001)]. Genotypes: (A, C) *Antp*^*NS-rvc12*^/*Antp*^*14*^, (B, D) *OregonR*, (E) *cas-Gal4/UAS-Antp*.

We examined Va-neuron identities in *Antp* mutants. The posterior prevalence rule predicts that Va-neuron neuropeptidergic identities would be unaffected, as Ubx would not be affected and posterior expansion of *Sex combs reduced* (*Scr*) would not confer neuropeptide identity on T1-T3 Va-neurons. Nonetheless, T3 Va-neurons in *Antp* mutants expressed DH31 and Dimm (Fig 5A and 5F), and Scr immunoreactivity did not expand posteriorly (S3 Fig).

We postulated that adoption of a Va-A1 terminal identity in the T3 segment was due to unanticipated anterior derepression of *Ubx* in the typical *Antp* domain. We examined Ubx expression in control T1-T3 Va-neurons, and found it at very low levels in T3 but not in T1 or T2 Va-neurons (Fig 5B). Low Ubx expression was maintained unaltered in T1-T3 Va-neurons in *Antp* mutants (Fig 5C). We thus conclude that loss of *Antp* removes a hindrance to Ubx function in T3 Va-neurons, which allows them to adopt a posterior A1-Va-type neuron fate, but does not interfere with Ubx expression. This violates the posterior prevalence rule that would favor posterior Ubx prevalence over anterior Antp function.

To determine whether this Antp repression of Va-DH31 in thoracic segments extends to other Va-neurons, we overexpressed *UAS-Antp* in Va-neurons using *cas-GAL4* and *elav-GAL4*. For *cas-Gal4*, we found downregulated expression of Capa (but not of DH31/AstA) (Fig 5D–5F); nonetheless, Antp did not downregulate Dimm. Following *Antp* ectopic expression in Va-neurons, we thus observed incomplete posterior expansion of T1-T3-type Va-neuronal fate in A2-A4 segments. There was no effect when *UAS-Antp* was expressed with *elav-Gal4* (S2C Fig). The data nonetheless indicate anterior prevalence of Antp over AbdA in postmitotic Va-neurons. To establish whether Antp downregulated AbdA directly, we analyzed and quantified AbdA immunoreactivity in A2-A4 Va-neurons after *cas-Gal4>UAS-Antp* overexpression (S4 Fig). AbdA levels were unaffected, which suggested that Antp acts on targets downstream of AbdA to decrease Capa expression.

## Discussion

**Fig 6:**
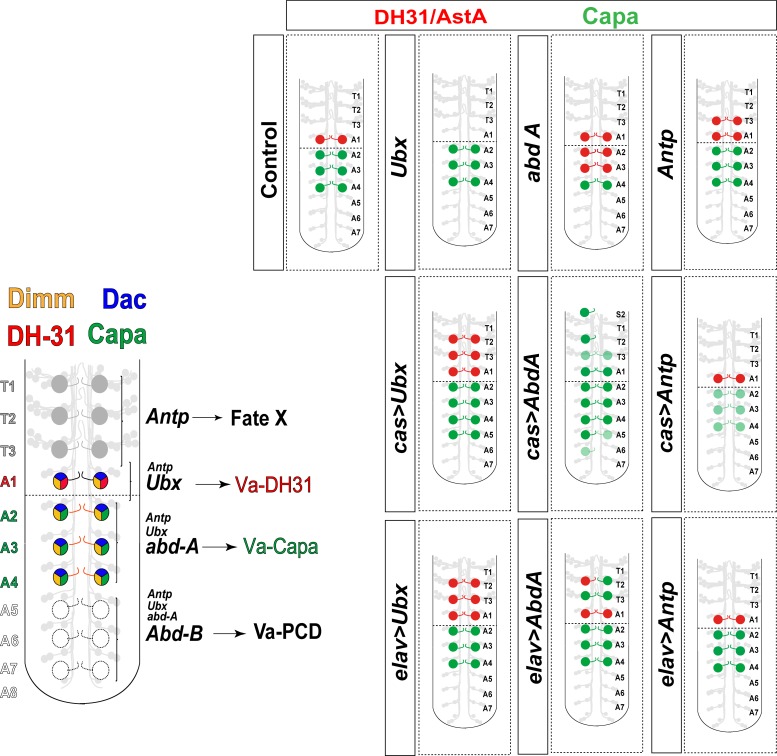
Specification of the segmentally homologous neurons. Model for how the segmentally homologous neurons acquire their final fate and cartoons summarizing the findings reported in this work.

Here we provide evidence that via segmental Hox gene activity, segmentally homologous neurons can be diversified into distinct neuropeptidergical identities. Va-A1 neurons terminally differentiate into DH31/AstA-expressing neurons (neuropeptidergic fate I) by virtue of *Ubx* activity, and Va-A2-A4 neurons terminally differentiate into Capa-expressing neurons (neuropeptidergic fate II) under the influence of *abdA*. In contrast, Va neurons born in abdominal segments A5-A8 are eliminated by PCD under the control of *Abd-B* (fate III) [8]. Va neurons thus provide an outstanding model with which to elucidate the mechanisms that underlie the diversification of segmentally homologous neurons (summarized in Fig 6).

### An alternative mechanism to produce neural segmental diversification in *Drosophila*

In the *Drosophila* VNC, segmental differences in neuronal identity along the AP axis can emerge via three general Hox gene-conducted mechanisms, 1) managing lineage size generated by the neuroblast [4, 5], 2) controlling the neuroblast proliferative mode in each segment [1, 4–6, 17, 18], or 3) driving or preventing PCD in postmitotic neurons in a segment-specific manner [7, 8, 10].

The third mechanism produces a highly refined expression pattern of a neuronal subtype whose logic is very simple: if a specific neuromere expresses a Hox gene that inhibits the PCD mechanism in a specific neuron type (of a series of segmentally homologous neurons), that neuron will be present in the neuromere. In contrast, if the role of the Hox gene is to carry out PCD, that neuromere will not exhibit that neuron.

In this study, we present a sophisticated alternative to the third mechanism, by which the Hox genes do not act merely as a death/life switch, but are responsible for a complete specification program that culminates in an alternative functional fate. A generic neuronal profile is generated along the AP axis. Distinct neural specification programs then act in a segment-specific manner to differentially specify mature cells, not as a binary choice through PCD control, but by managing the two alternative functional neuropeptidergic fates in these neurons. *Ubx* action is responsible for Va-DH31 neuron specification (DH-31 neuropeptide-expressing), whereas *abdA* specifies other neurons in the series as Va-Capa (Capa neuropeptide-expressing).

These findings demonstrate that segmentally homologous neurons can undergo three distinct segment-specific programs for cell subtype specification (PCD and two neuropeptidergic fates). It is tempting to speculate that mechanisms similar to this alternative in the Drosophila nervous system might exist in other animal lineages. Our findings are consistent with previous observations of the developing vertebrate neural tube, which showed that Hox genes can also control the identity of early postmitotic neurons, specifying different spinal motor neuron subtypes [19]. In that case, however, there was no evidence that the motor neurons are generated by the same neuroblast and born in the same temporal window, and they might not be segmentally homologous. It will thus be of interest to identify additional cases of Hox gene switching of functional cell fates and to unmask the underlying mechanisms.

Here we provide evidence as to how segmentally homologous neurons acquire two different terminal neuropeptidergic fates in the Drosophila nervous system. In addition, Va neurons generated in segments A5-A8 undergo a PCD process, whereas the fate of Va neurons born in the thoracic segment remains obscure. This precise expression pattern is achieved through the intricate interplay of Hox genes, which shape the segment-specific structures of the nervous system. Such complexity presumably underlies specific physiological output, although the physiological basis of this differentiation program remains unknown. Future studies must define the innervation of all Va neurons (or least of Va-Capa and Va-DH31) to analyze the phenotypes produced by their specific ablation and by specific DH1/AstA and Capa silencing in these cells (to block function of DH31/AstA/Capa-expressing neurons in the CNS). Studies are also needed to address the fate of Va neurons born in the thoracic segments.

In summary, we have established the identity of the Va neuron born in the A1 segment and have shown that newborn segmentally homologous neurons can acquire different neuropeptide fates by undergoing specific-segment programs of neural subtype specification. Although lack of segment-specific markers to identify diversity amongst synonymous neurons currently limits our ability to address this question, we suspect that similar mechanisms operate in other *Drosophila* CNS lineages. The alternative mode by which Hox genes shape *Drosophila* segmental neural architecture, more complex than the binary “live” or “not live” mechanism, broadens our knowledge of the processes involved in diversifying neuronal identities within the CNS.

## Experimental procedures

### Fly stocks

Fly stocks were raised and crosses performed at 25°C on standard medium. The following fly mutant alleles were used: *elav-Gal4*, *castor-Gal4*, *UAS-Antp*, *UAS-Ubx*, *UAS- abdA*, *Ubx*^*1*^, *Antp*^*Ns-rvC12*^, *Antp*^*14*^, *abdA*^*MX1*^, *UAS-dimm* (provided by S. Thor, Linköping University, Sweden). Mutants were maintained on *CyO*, *Act-GFP*; *CyO*, *Dfd-EYFP*; *TM3*, *Ser*, *Act-GFP*; *CyO*, *twi-Gal4*, *UAS-GFP*; *TM3*, *Sb*, *Ser*, *twi-Gal4*, *UAS-GFP*; or *TM6*, *Sb*, *Tb*, *Dfd-EYFP* balancer chromosomes. As wild type, *Oregon-R* was usually used. Unless otherwise stated, flies were obtained from the Bloomington Drosophila Stock Center (Bloomington, IN, USA).

### Immunohistochemistry

The antibodies used were guinea pig anti-Dimm (1:1000, provided by S. Thor; Baumgardt et al., 2007), rabbit anti-Capa, -DH31, -AstA (1:1000; all provided by J. Veenstra, Université de Bordeaux I, France), mAb anti-Dac (1:25, from Developmental Studies Hybridoma Bank, Iowa City, IA, USA). All polyclonal sera were preabsorbed against pools of early embryos. Secondary antibodies were conjugated with fluorescein isothiocyanate, rhodamine-RedX or Cy5, and used at 1:500 (Jackson ImmunoResearch, West Grove, PA, USA). Embryos were dissected in PBS, fixed in 4% paraformaldehyde (25 min), blocked, and processed with antibodies in PBS with 0.2% Triton-X100 and 4% donkey serum. Slides were mounted with Vectashield (Vector, Burlingame, CA, USA). In all cases, wild type and mutant embryos were stained and analyzed on the same slide.

### Confocal imaging, data acquisition and staining quantification

A Zeiss META 510 confocal microscope was used to collect data for all fluorescent images; confocal stacks were merged using Zeiss LSM software or Adobe Photoshop CS4. Where appropriate, images were false-colored to assist color-blind readers or to represent data more clearly.

### Statistical methods

Statistical analysis was performed using Microsoft Excel. Quantifications of observed phenotypes were performed using Student’s two-tailed t test, assuming equal variance.

## Supporting information

S1 Fig Va-A1 neurons survive into larval stages. Expression of Dimm (green) and Dac (red) in wild type at larval stage III. Genotype: *OregonR*.
(PDF)

S2 Fig Gain-of-function experiments using the *elav-Gal4* driver. Expression of Dimm (green) and DH31 (red) in *elav>Ubx* (A), *elav>abdA* (B) and *elav>Antp* (C). (D) Quantitation of genetic studies [*n* ≥8 VNC in all genotypes; asterisks indicate significant difference compared with control (Student’s *t*-test, *P*<0.001)]. Genotypes: (A) *elav-Gal4*/*UAS-Ubx*, (B) *elav-Gal4*/*UAS-abdA*, (C) *elav-Gal4*/*UAS-Antp*, (D) *OregonR* and as in (A-C).
(PDF)

S3 Fig Scr (Sex combs reduced) immunoreactivity did not expand posteriorly. Expression of Dimm (green) and Scr (red) in wild type and *Antennapedia* mutants. Genotypes: (A) *Antp*^*NS-rvc12*^/*Antp*^*14*^ and (B) *OregonR*.
(PDF)

S4 Fig AbdA levels are unaffected in by *cas-Gal4>UAS-Antp* overexpression. (A, B) Expression of Capa (green) and AbdA (red) in controls (A) and *cas>Antp* (B). (C) Quantitation of genetic studies [*n* ≥8 VNC]. Genotypes: (A) *OregonR*, (B) *cas-Gal4/UAS-Antp*
(PDF)
