# Supplementary figures and images for: Segmentally homologous neurons acquire two different terminal neuropeptidergic fates in the Drosophila nervous system
Source: PLoS One. 2018 Apr 10;13(4):e0194281. doi: 10.1371/journal.pone.0194281 (PMC5892886; doi:10.1371/journal.pone.0194281)

# Figure S1

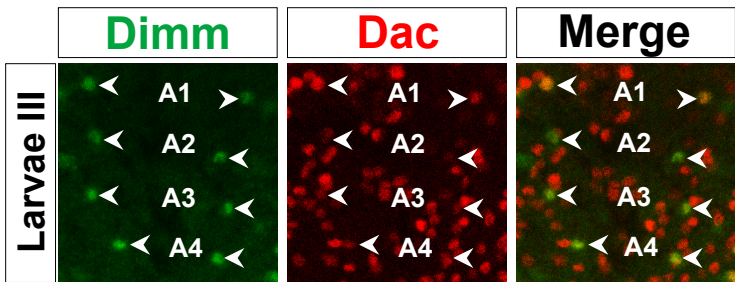

Supplement: S1 Fig — Expression of Dimm (green) and Dac (red) in wild type at larval stage III. Genotype: OregonR. (PDF) [file pone.0194281.s001.pdf]

# Figure S2

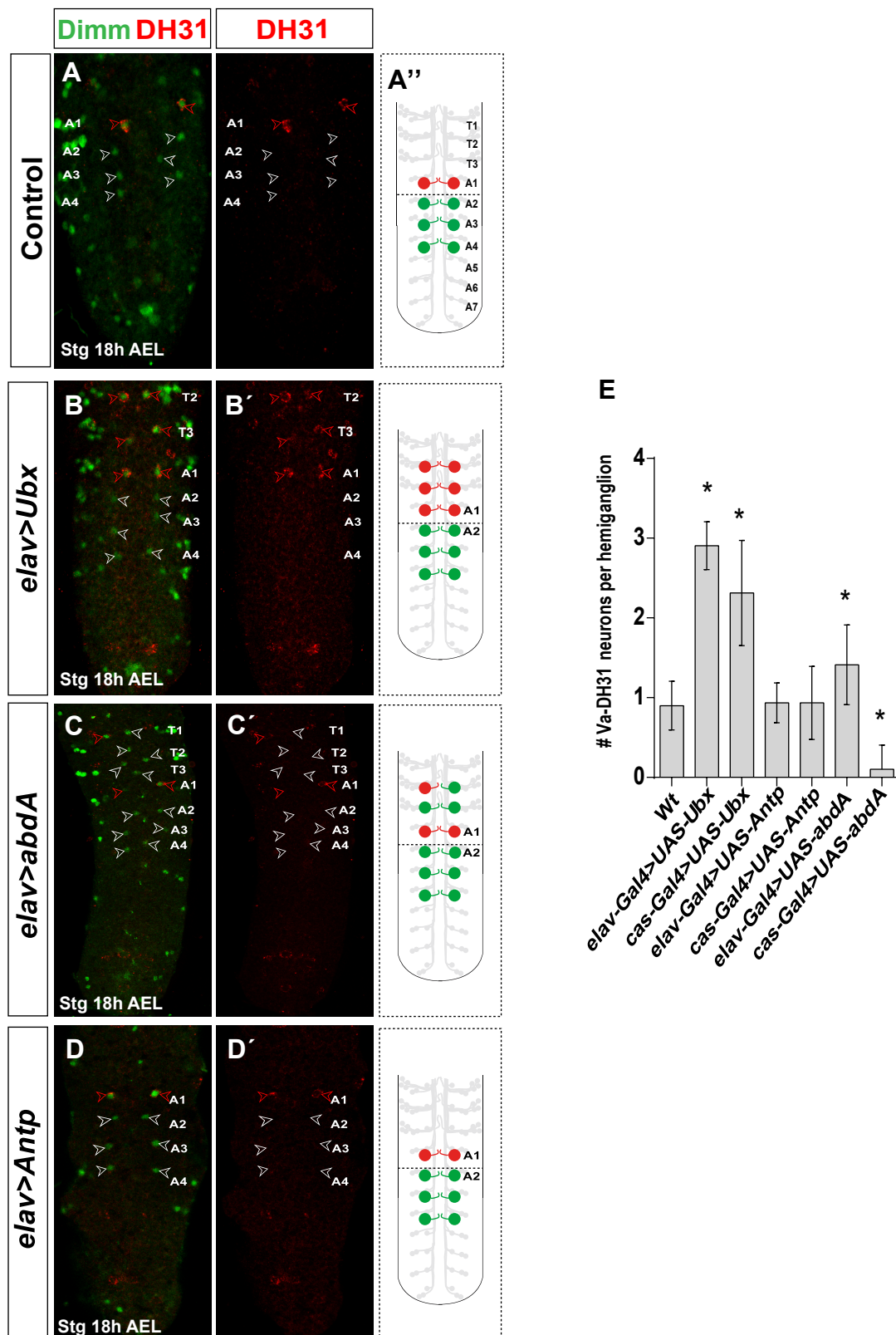

Supplement: S2 Fig — Expression of Dimm (green) and DH31 (red) in elav>Ubx (A), elav>abdA (B) and elav>Antp (C). (D) Quantitation of genetic studies [n ≥8 VNC in all genotypes; asterisks indicate significant difference compared with control (Student’s t-test, P<0.001)]. Genotypes: (A) elav-Gal4/UAS-Ubx, (B) elav-Gal4/UAS-abdA, (C) elav-Gal4/UAS-Antp, (D) OregonR and as in (A-C). (PDF) [file pone.0194281.s002.pdf]

Figure S3

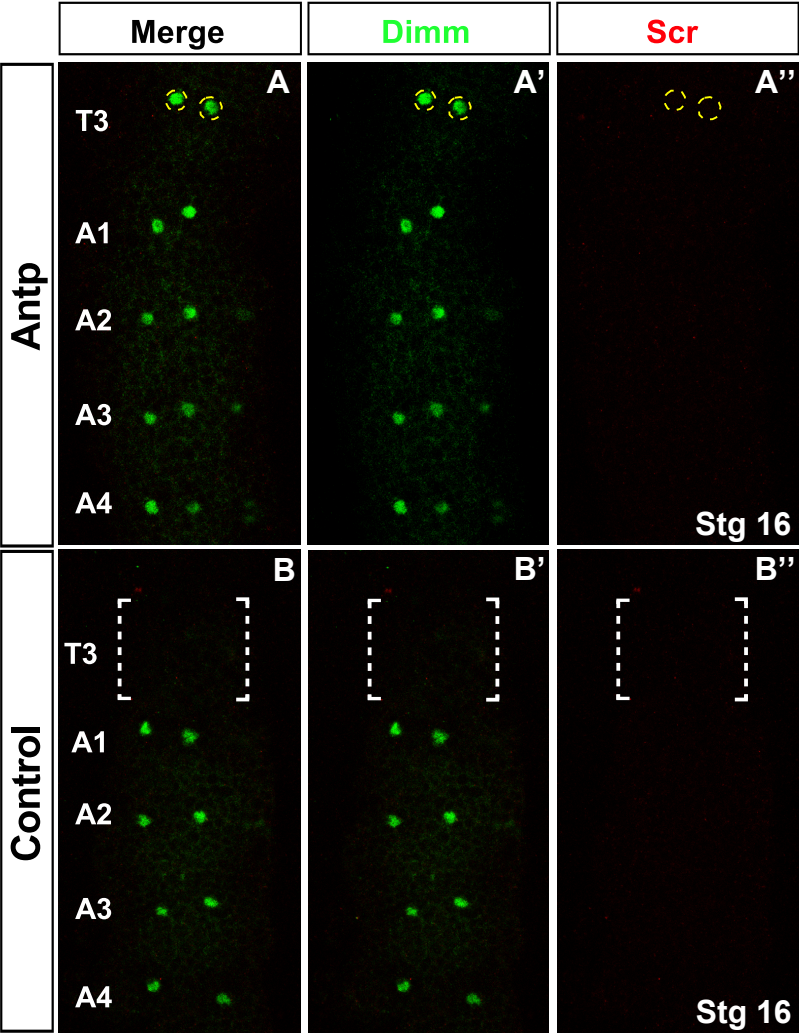

Supplement: S3 Fig — Expression of Dimm (green) and Scr (red) in wild type and Antennapedia mutants. Genotypes: (A) AntpNS-rvc12/Antp14 and (B) OregonR. (PDF) [file pone.0194281.s003.pdf]

Figure S4

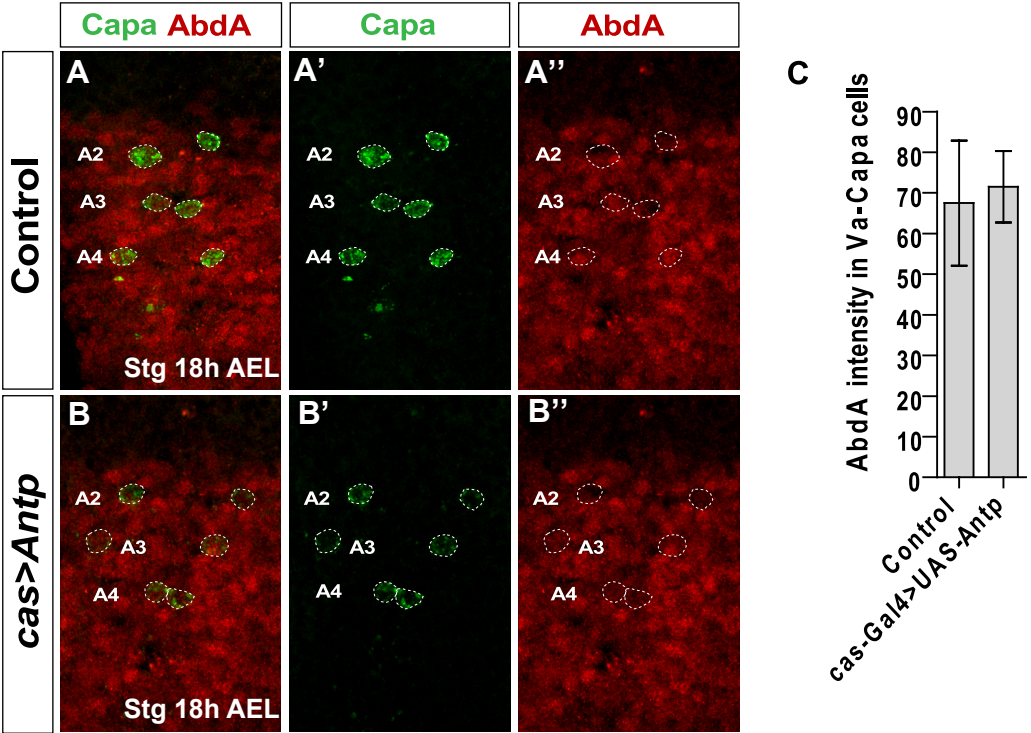

Supplement: S4 Fig — (A, B) Expression of Capa (green) and AbdA (red) in controls (A) and cas>Antp (B). (C) Quantitation of genetic studies [n ≥8 VNC]. Genotypes: (A) OregonR, (B) cas-Gal4/UAS-Antp (PDF) [file pone.0194281.s004.pdf]
